# Diagnostic accuracy for CZT gamma camera compared to conventional gamma camera technique with myocardial perfusion single-photon emission computed tomography: Assessment of myocardial infarction and function

**DOI:** 10.1007/s12350-022-03185-0

**Published:** 2023-03-13

**Authors:** Fredrik Hedeer, Shahnaz Akil, Jenny Oddstig, Cecilia Hindorf, Håkan Arheden, Marcus Carlsson, Henrik Engblom

**Affiliations:** 1 Department of Clinical Physiology, Skåne University Hospital, Lund University, Lund, Sweden; 2 Radiation Physics, Department of Hematology, Oncology and Radiation Physics, Skåne University Hospital, Lund, Sweden

**Keywords:** Myocardial ischemia and infarction, MRI, Gated SPECT, MPI, CAD, SPECT

## Abstract

**Background:**

The solid-state cadmium-zinc-telluride (CZT) gamma camera for myocardial perfusion single-photon emission computed tomography (MPS) has theoretical advantages compared to the conventional gamma camera technique. This includes more sensitive detectors and better energy resolution. We aimed to explore the diagnostic performance of gated MPS with a CZT gamma camera compared to a conventional gamma camera for detection of myocardial infarct (MI) and assessment of left ventricular (LV) volumes and ejection fraction (LVEF), using cardiac magnetic resonance (CMR) as the reference method.

**Methods:**

Seventy-three patients (26% female) with known or suspected chronic coronary syndrome were examined with gated MPS using both a CZT gamma camera and a conventional gamma camera as well as with CMR. Presence and extent of MI on MPS and late gadolinium enhancement (LGE) CMR was evaluated. For LV volumes, LVEF and LV mass, gated MPS images and cine CMR images were evaluated.

**Results:**

MI was found in 42 patients on CMR. The overall sensitivity, specificity, positive and negative predictive values for the CZT and the conventional gamma camera were the same (67%, 100%, 100% and 69%). For infarct size > 3% on CMR, the sensitivity was 82% for the CZT and 73% for the conventional gamma camera, respectively. LV volumes were significantly underestimated by MPS compared to CMR (*P* ≤ .002 for all measures). The underestimation was slightly less pronounced for the CZT compared to the conventional gamma camera (2-10 mL, *P* ≤ .03 for all measures). For LVEF, however, accuracy was high for both gamma cameras.

**Conclusion:**

Differences between a CZT and a conventional gamma camera for detection of MI and assessment of LV volumes and LVEF are small and do not appear to be clinically significant.

**Supplementary Information:**

The online version contains supplementary material available at 10.1007/s12350-022-03185-0.

## Introduction

Gated myocardial perfusion single-photon emission computed tomography (MPS) can be used to detect the presence of a myocardial infarct (MI) as well as assess left ventricular (LV) volumes and ejection fraction (LVEF). Previous studies have shown that MPS has limited ability to detect MI’s, especially small subendocardial infarcts, compared to the reference method late gadolinium enhancement cardiac magnetic resonance (LGE-CMR).^1–3^ Furthermore, MPS systematically underestimates LV volumes compared to the reference method CMR, however, accuracy differs between different MPS software.^4–7^

One of the main reasons behind the limited diagnostic performance of MPS, is the limited spatial resolution associated with the SPECT technique using the conventional Anger scintillation gamma camera with NaI crystal detectors and parallel hole collimators. In recent years, a new generation of gamma camera systems has evolved utilizing a solid-state detector technique with cadmium-zinc-telluride (CZT) and pin hole collimators, resulting in improved spatial resolution, energy resolution and count sensitivity compared to the conventional gamma cameras. Thus, this technique could potentially improve the diagnostic performance of MPS.^8–10^ However, the diagnostic performance of gated MPS with a CZT gamma camera compared to a conventional gamma camera for detection of MI and assessment of LV volumes and EF, using CMR as the reference method, has to our knowledge not yet been investigated.

The aim of this study was to explore the diagnostic performance of gated MPS with a dedicated cardiac CZT gamma camera compared to a dedicated cardiac conventional gamma camera for detection of MI and assessment of LV volumes and LVEF, using CMR as the reference method.

## Methods

### Study Population and Design

The study protocol was approved by the Regional Ethics Committee at Lund University (LU2013/550 and LU2013/4010). Patients were included in two ways. (1) Patients clinically referred for CMR imaging, where CMR images showed evidence of ischemic scar were asked to participate in the study. The patients were examined with MPS at rest and images were acquired in two gamma cameras, a CZT gamma camera and a conventional gamma camera. Out of 47 included patients, 7 were excluded because SPECT data for both gamma cameras could not be obtained due to intermittent technical problems with the scanner table on the conventional gamma camera, one was excluded because of inadequate LGE-CMR image quality, one was excluded because CMR was performed during the acute phase of the MI and two were excluded because of presence of left bundle branch block which is known to possibly affect the MPS image uptake pattern.^11^ Thus, 36 patients could be used for image analysis of both MPS and CMR. (2) In addition, a subset of the patients was recruited from another study (the MYOMER study), in which patients clinically referred for an elective coronary angiography (CA) because of known or suspected chronic coronary syndrome were included. The goals with the MYOMER study were to study myocardial perfusion imaging before and after CA, with or without percutaneous coronary intervention. From the MYOMER study, 37 patients were examined with CMR and MPS at rest with image acquisition in both gamma cameras before the CA examination, and therefore could be included in the current study. Thus, in total the study population consisted of 73 patients with two MPS image acquisitions each resulting in 146 datasets that were evaluated. Patient charts were reviewed for patient characteristics and to exclude any cardiac adverse event, coronary revascularization or changes in cardiac medication occurring between the CMR and MPS examinations.

### MPS

#### Image acquisition

Patients were examined at rest and injected with a weight adjusted activity of 4 MBq·kg^−1^ of ^99m^Tc-tetrofosmin (GE Healthcare) (356 ± 69 MBq). Image acquisition was performed 45-60 minutes after the injection. Each patient was examined in both supine and prone position and in both a cardiac dedicated CZT gamma camera (Discovery NM 530c, GE Healthcare) and a cardiac dedicated conventional gamma camera (Ventri, GE Healthcare). There was no systematic order in which gamma camera was used for the first and second image acquisition, since image acquisition of the study patients had to be accommodated to the clinical flow of patients.

The acquisition time on the CZT gamma camera was 480 seconds. The images were reconstructed with a Maximum Likelihood Estimation Method (MLEM) algorithm, 40 iterations; Green OSL regularization α parameter of 0.51 and a β of 0.3 and post filtered with a Butterworth filter with a cut-off frequency of 0.37 and a power of 7. For the conventional gamma camera the examination was performed with the detectors in L-mode. Sixty projections were acquired in a total angular range of 180° with a stop condition of 25 seconds per projection. The conventional gamma camera images were reconstructed with a resolution recovery OSEM algorithm (Evolution, GE Healthcare) using 12 iterations and 10 subsets and post filtered with a Butterworth filter with a cut-off frequency of 0.4 and a power of 10. All reconstruction parameters used followed recommendations from the manufacturer. The reconstructed images were reformatted to the standard cardiac axis format (short-axis, vertical long-axis and horizontal long-axis). ECG-gated image acquisition using 8 frames per cardiac cycle was performed for all supine acquisitions. ECG-triggering failed in two acquisitions for the conventional gamma camera, due to poor ECG signal. Attenuation correction was not applied.

#### MPS image analysis

All MPS images were analyzed using the software Segment, version 2.2 (Medviso AB, Lund, Sweden) and QGS/QPS, version 2015.6 (Cedars-Sinai, Los Angeles, USA). For LV volumes, EF and mass the software were used following recommendations from the manufacturers. Briefly, both reconstructed static and gated images were analyzed by fully automated LV segmentation algorithms. LV end-diastolic volume (LVEDV), LV end-systolic volume (LVESV), LV stroke volume (LVSV) and LVEF were calculated from the gated images and LV mass (LVM) was calculated from the static images. Manual correction was performed if the automatic segmentation was obviously wrong. For assessment of MI, gated and summed MPS images, acquired in supine position, were loaded into the QGS/QPS software. One experienced observer, blinded to patient data, visually evaluated the images in random order as previously described.^3^ Briefly, a perfusion defect in the summed images with decreased wall thickening in the gated images was reported as MI. If the observer felt uncertain after evaluating the images acquired in supine position, the summed images acquired in both supine and prone position were used. Infarcts were located to the left anterior descending artery (LAD) territory (anterior, septal and/or apical parts of the LV) or to the left circumflex artery/right coronary artery (LCx/RCA) territory (lateral and/or inferior parts of the LV). Additionally, regional myocardial tracer uptake in each LV segment according to the standardized 17 segments model^12^ was quantified using a 5-point scale ranging from 0 (normal uptake) to 4 (absent uptake), where regional motion according to the gated images was taken into account. Thus, for a segment to be scored as reduced uptake, regional motion in that segment would have to be affected. If uptake was judged to be reduced but motion in that segment was judged to be normal, the score was set to 0. The total score of the left ventricle at rest, summed rest score (SRS) was calculated. Twenty cases were evaluated twice and by a second observer to calculate intra- and interobserver variability for infarct detection. For both the CZT and the conventional gamma camera, epi- and endocardial borders were derived from automated delineation provided by the MPS software. Therefore, intra- and interobserver variability for functional parameters by MPS were not assessed.

### CMR

#### Image acquisition

CMR imaging was performed on a Philips Intera CV (Best, The Netherlands) for seven patients, on a Siemens Magnetom Aera (Erlangen, Germany) for 63 patients and on a Siemens Magnetom Avanto (Erlangen, Germany) for three patients. All subjects were placed in supine position. Cine short-axis gradient-recalled echo images covering the left ventricle were acquired using a balanced turbo field echo (bTFE) sequence: slice thickness = 8 mm, field-of-view = 340 mm, TR = 3.14 ms, TE = 1.58 ms. Three cine long-axis images (2-, 3- and 4-chamber views) were acquired using the same sequence. Approximately 15 min after intravenous administration of an extracellular gadolinium-based contrast agent (gadoteric acid, Gd-DOTA, 0.2 mmol·kg^−1^, Guerbet, Gothia Medical AB, Billdal, Sweden) an inversion-recovery (IR) sequence was used to acquire late gadolinium enhanced (LGE) images in the corresponding planes as for the cine images. Typical LGE sequence parameters were: slice thickness = 8 mm, TR = 3.9 ms, TE = 1.2 ms, in-plane resolution = 1.5 × 1.5 mm and flip angle = 15º with acquisition every heartbeat. The inversion time, typically 250-350 ms, was manually adjusted to null the signal from remote myocardium.

#### CMR image analysis

All CMR images were analyzed using the software Segment, version 2.2. The endo- and epicardium of the LV were manually delineated in the cine short-axis images in both end-diastole and end-systole by two observers in consensus. The LV end-diastole and end-systole were defined as the time frame with the largest and the smallest LV blood pool volume, respectively. Trabecular and papillary muscles not contiguous with the myocardial wall were excluded, thus included in the LV cavity volume. The endo- and epicardial borders were adjusted between end-diastole and end-systole to accomplish the same LVM in both time frames. Based on the LV delineation, LVEDV, LVESV, LVSV and LVEF were calculated. LVM was calculated as the muscle volume between the endo- and epicardial delineations, multiplied by the density of the myocardium (1.05·g·mL^−1^).^13^ Assessment of MI was performed on the LGE-CMR images, where hyperenhanced regions extending from the LV endocardium in two perpendicular imaging planes according to typical coronary artery territories, were considered MI. MI’s were visually located to the LAD or the LCx/RCA territory. MI size was quantified using a semi-automatic method, the EWA algorithm, as previously described with manual corrections if needed.^14^ Infarct transmurality was quantified as the infarct extension measured from the endocardium to the epicardium, both per segment and for the over-all infarct (mean transmurality).

### Statistical Analysis

Data are presented as mean ± SD or median (interquartile range 25%-75%) unless otherwise stated. All statistical calculations were performed using either Prism 7.04 (GraphPad Software, San Diego, CA, USA) or Microsoft Excel 2013 (Microsoft Corporation, Redmond, WA, USA). LV volumes, EF and mass by MPS and CMR were compared using Student’s *t*-test. The absolute differences between MPS and CMR for LV volumes, EF and mass were investigated with modified Bland-Altman analysis, using the reference method CMR on the *x*-axis and the absolute difference between MPS and CMR on the *y*-axis. A *P*-value of < .05 was considered to indicate statistical significance.

## Results

Patient characteristics are presented in Table 1. Median time between CMR and MPS examinations was 6 (1-56) days. None of the patients had signs of cardiac events, had any coronary revascularization or had changes in cardiac medication during the time between CMR and MPS examinations. Mean heart rate during MPS imaging was 64 ± 9 beats·min^−1^ and during CMR imaging 65 ± 10 beats·min^−1^ (*P* = .09). For gamma camera imaging, 37 patients were scanned in the CZT gamma camera first, while 36 patients were scanned in the conventional gamma camera first.

**Table 1:** Patient characteristics

Age (years)	66 ± 10
Female	19 (26%)
BMI (kg·m^−2^)	27 ± 4
Risk factors	
Hypertension	48 (66%)
Diabetes	17 (23%)
Hyperlipidemia	47 (64%)
Current or former smoker	42 (57%*)
Family history of CAD	17 (25%*)
Suspected previous MI	34 (47%*)
Clinical diagnosis of heart failure	8 (11%)
Previous CABG	7 (9%)
Previous PCI	27 (37%)
Medications	
Anticoagulants	66 (90%)
Beta-blockers	43 (59%)
ACE inhibitor/ARBs	52 (71%)
Statins	65 (89%)

Data are presented as mean ± SD or absolute number (proportion in %)

*BMI*, body mass index; *CAD*, coronary artery disease; *MI*, myocardial infarct; *CABG*, coronary artery bypass grafting; *PCI*, percutaneous coronary intervention; *ACE*, angiotensin-converting enzyme; *ARB*, angiotensin II receptor blocker

*Information about smoking history and suspected previous MI was obtained for 72 and about family history of CAD for 66 out of 73 patients

**Figure 1:**
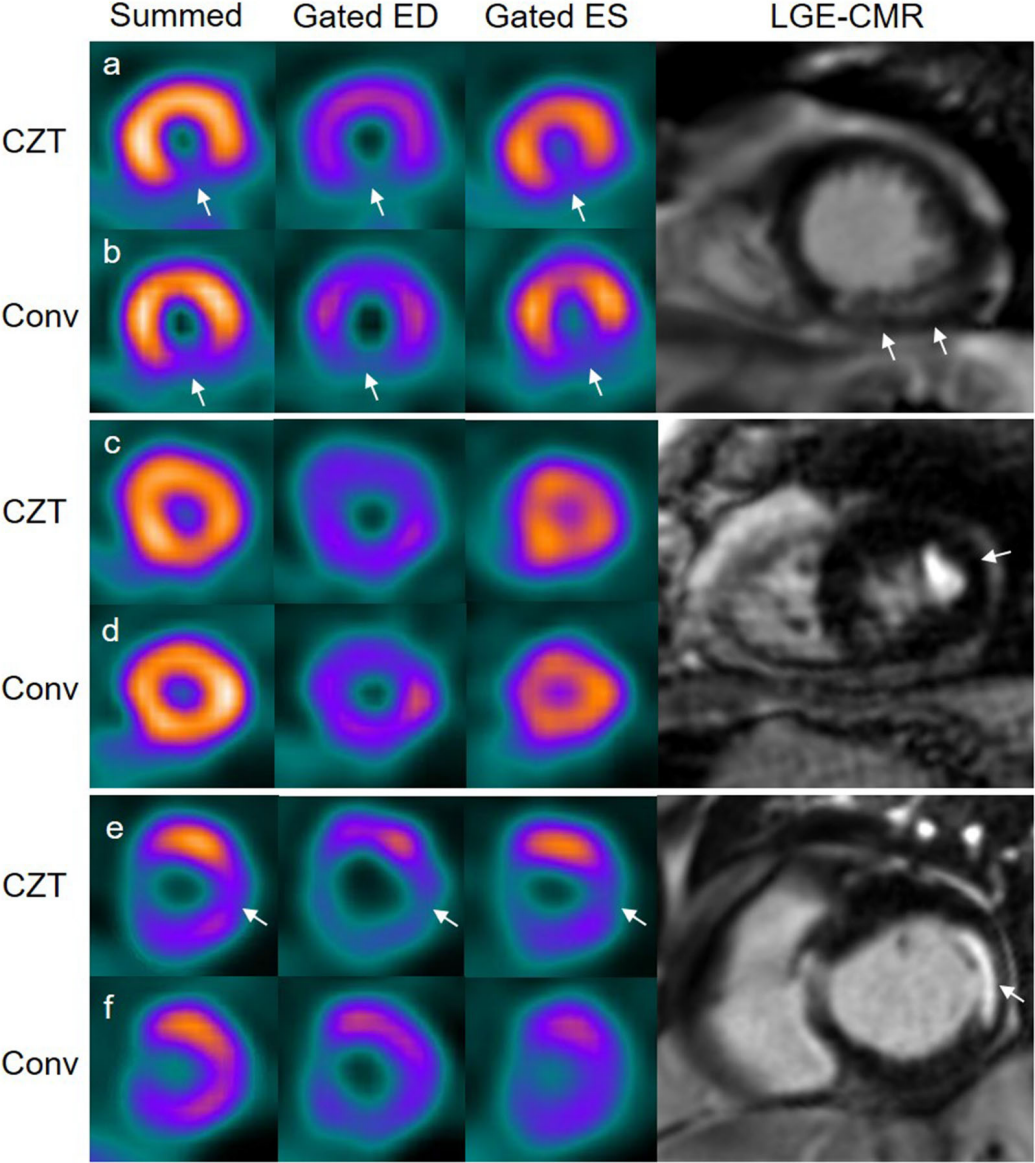
Patient examples of myocardial infarct (MI) by cardiac magnetic resonance (CMR), myocardial perfusion SPECT (MPS) with a cadmium-zinc-telluride (CZT) gamma camera and a conventional (Conv) gamma camera. Columns from left to right show summed MPS perfusion images, gated MPS in end-diastole (ED) and end-systole (ES) and late gadolinium enhancement (LGE)-CMR. Case *a* and *b* are examples of an MI in the apical inferior wall on CMR which is correctly diagnosed by both CZT and conventional MPS (arrows). Case *c* and *d* are examples of an MI in the apical lateral wall on CMR (arrow), missed by both CZT and conventional MPS. Case *e* and *f* are examples of an MI in the basal lateral wall on CMR correctly diagnosed by CZT MPS (arrows) but missed by conventional MPS

MIs were found in 42 patients on CMR, in 28 patients on MPS with the CZT gamma camera and in 28 patients on MPS with the conventional gamma camera. On a patient level, no patients were found to have MI on MPS but not on CMR, neither for the CZT nor for the conventional gamma camera (Tables 2 and 3). On a patient basis, sensitivity, specificity, positive predictive value (PPV) and negative predictive value (NPV) were 67%, 100%, 100% and 69% on MPS with both the CZT and the conventional gamma camera. The number of patients with MI in each coronary artery territory on CMR and MPS are shown in Table 4. On a vessel territory basis, there were no false positive MI cases on MPS with the CZT gamma camera. Sensitivity, specificity, PPV and NPV for the CZT gamma camera on a vessel territory basis were 54%, 100%, 100% and 91% for the LAD territory and 66%, 100%, 100% and 76% for the LCx/RCA territory. On a vessel territory basis, 3 patients were found to have infarcts in the LAD territory and 1 patient in the LCx/RCA territory on MPS with the conventional gamma camera but not on CMR. Sensitivity, specificity, PPV and NPV for the conventional gamma camera on a vessel territory basis were 38%, 95%, 63% and 88%, respectively, for the LAD territory and 63%, 97%, 96% and 74%, respectively, for the LCx/RCA territory. Patient examples are shown in Figure 1.

**Table 2:** Number of patients with myocardial infarct (MI) on CMR and MPS with CZT gamma camera imaging

	MI on CMR	No MI on CMR
MI on MPS CZT	28	0
No MI on MPS CZT	14	31

*CMR*, cardiac magnetic resonance; *MPS*, myocardial perfusion SPECT; *CZT*, cadmium-zinc-telluride

**Table 3:** Number of patients with myocardial infarct (MI) on CMR and MPS with conventional gamma camera imaging

	MI on CMR	No MI on CMR
MI on conventional MPS	28	0
No MI on conventional MPS	14	31

*CMR*, cardiac magnetic resonance; *MPS*, myocardial perfusion SPECT

**Table 4:** Number of patients with MI in each coronary artery territory on CMR and MPS

	LAD	LCx/RCA	LAD and LCx/RCA
CMR	7	29	6
CZT MPS	5	21	2
Conventional MPS	5	20	3

*MI*, myocardial infarct; *CMR*, cardiac magnetic resonance; *MPS*, myocardial perfusion SPECT; *CZT*, cadmium-zinc-telluride; *LAD*, left anterior descending artery; *LCx*, left circumflex artery; *RCA*, right coronary artery

For intra-observer variability, twenty cases were evaluated twice for presence of MI or not on a per patient level, showing agreement in 19 out of 20 cases. For inter-observer variability, the same twenty cases were evaluated by a second observer for presence of MI or not on a patient level, showing agreement in 16 out of 20 cases.

**Figure 2:**
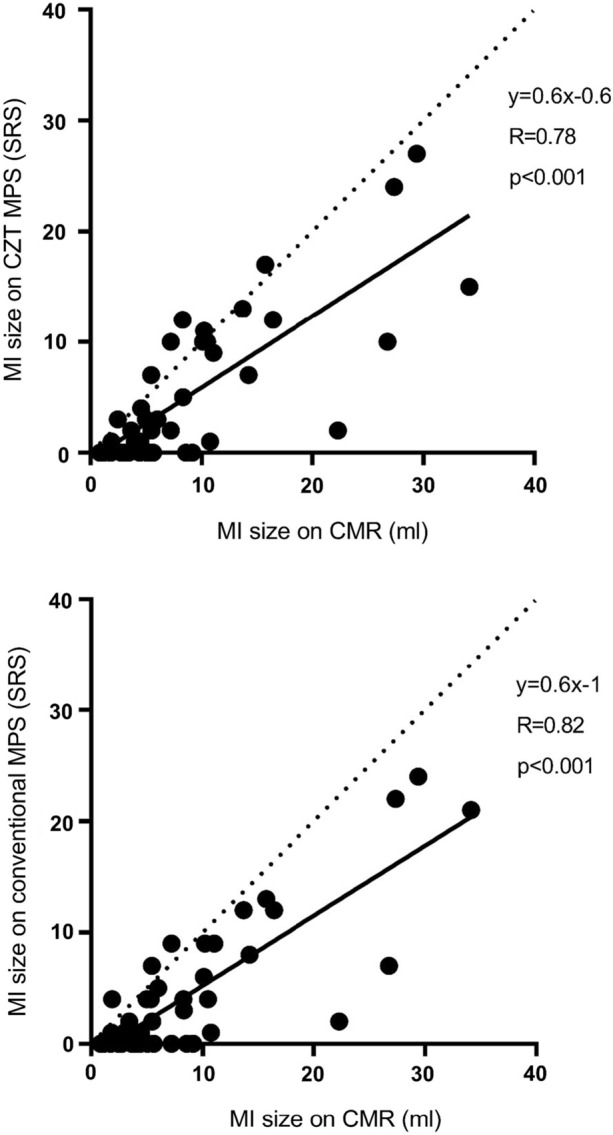
Correlation between MI size on CMR in mL compared to MI size on MPS by summed rest score (SRS) for the solid-state CZT detector gamma camera and the conventional gamma camera, respectively. The solid line indicates the regression line and the dashed line indicates line of identity. *MI*, myocardial infarct; *CMR*, cardiac magnetic resonance; *MPS*, myocardial perfusion SPECT; *CZT*, cadmium-zinc-telluride; *SRS*, summed rest score

Infarct size on CMR expressed as volume, % of the LV and transmurality is shown in Table 5. Figure 2 shows the agreement between MI size on CMR in ml compared to MI size on MPS by SRS. Using a cut-off value for infarct size of 3% of LV, the sensitivity on a patient level was 82% and 73% on MPS with the CZT gamma camera and the conventional gamma camera, respectively, with unchanged specificity. Using a cut-off value for infarct size of 10% of LV, the sensitivity on a per patient level was 100% on MPS for both gamma cameras. Based upon mean MI transmurality on CMR, the 42 MI patients were divided into two halves: the 21 patients with lowest mean MI transmurality (ranging from 25% to 42% mean MI transmurality) and the 21 patients with highest mean MI transmurality (ranging from 43% to 61% mean MI transmurality). In the patient group with lowest mean MI transmurality, 11 out of the 21 patients were correctly diagnosed with MPS CZT gamma camera and 13 out of the 21 patients were correctly diagnosed with MPS conventional gamma camera. In the patient group with highest mean MI transmurality, 17 out of the 21 patients were correctly diagnosed with MPS CZT gamma camera and 15 out of the 21 patients were correctly diagnosed with MPS conventional gamma camera.

**Table 5:** Myocardial infarct (MI) size on CMR in the 42 patients with (+) or without (−) perfusion defects on MPS

	CMR MI size (% of LV)	CMR mean MI transmurality (%)
CZT MPS + (n = 28)	10 ± 7	47 ± 10
Conventional MPS + (n = 28)	10 ± 7	46 ± 11
CZT MPS − (n = 14)	4 ± 2	37 ± 8
Conventional MPS − (n = 14)	4 ± 2	40 ± 9

Data are presented as mean ± SD

*CMR*, cardiac magnetic resonance; *MPS*, myocardial perfusion SPECT; *CZT*, cadmium-zinc-telluride; *LV*, left ventricle. + Denotes patients correctly diagnosed with MI on MPS, − denotes MI patients that were false negative on MPS

In 13 examinations, the observer used summed MPS images acquired in both supine and prone position. In one case with a small MI on CMR (1.4% of the LV), the MPS CZT gamma camera diagnosis was changed from MI to no MI after the use of prone images. In two cases with no MI on CMR, the MPS conventional gamma camera diagnosis was changed from MI in the inferior wall to no MI after the use of prone images. Diagnosis was unchanged for the other 10 cases.

**Figure 3:**
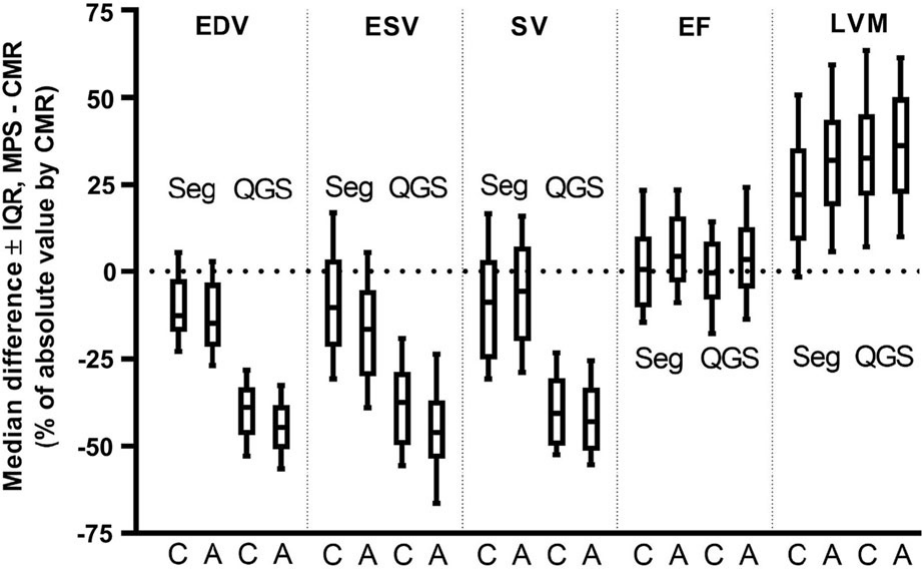
Percent median bias ± interquartile range 10%-90% (IQR) for MPS compared to absolute values by CMR for left ventricular end-diastolic volume (EDV), left ventricular end-systolic volume (ESV), left ventricular stroke volume (SV), left ventricular ejection fraction (EF) and left ventricular mass (LVM). Results are shown for both the solid-state CZT detector gamma camera (C) and the conventional Anger gamma camera (A) and for the two MPS software Segment (Seg) and QGS/QPS (QGS). MPS bias compared to CMR was significant for all measurements for both gamma cameras and both software (*P* ≤ .002 for all comparisons). Bias compared to CMR differed significantly between the CZT and conventional gamma camera for all measurements (*P* ≤ .03 for all measurements by Segment software and *p* ≤ .01 for all measurements by QGS/QPS software). *CMR*, cardiac magnetic resonance; *MPS*, myocardial perfusion SPECT

Table 6 shows the results of LVEDV, LVESV, LVSV, LVEF and LVM for CMR and MPS comparing the CZT and the conventional gamma cameras. MPS significantly underestimated LV volumes and overestimated LVM, for both gamma cameras and MPS software, compared to CMR (*P* ≤ .002 for all comparisons). LVEF did not differ between CMR and MPS with the CZT gamma camera (*P* = .85 and .82 for Segment and QGS/QPS software, respectively), while a significant overestimation of LVEF was shown for MPS with the conventional gamma camera compared to CMR (*P* = .001 and < .05 for Segment and QGS/QPS software, respectively). Comparing MPS software, there were significant differences for LV volumes and LVM between Segment and QGS/QPS (*P* < .001 for all parameters), while no significant difference was shown for LVEF (*P* = .95 and .09 for CZT and conventional gamma camera, respectively). Bias of LV parameters for MPS compared to absolute values by CMR are shown in Figure 3. Bias compared to CMR differed significantly between the CZT and conventional gamma camera for all measurements (*P* ≤ .03 for all measurements by Segment software and *P* ≤ .01 for all measurements by QGS/QPS software).

**Table 6:** Left ventricular volumes, ejection fraction and mass by MPS and CMR

	MPS Segment software		MPS QGS/QPS software		CMR
	CZT	Conventional	CZT	Conventional	
LVEDV (mL)	170 ± 60	167 ± 60*	117 ± 48	107 ± 45^†††^	189 ± 57
LVESV (mL)	81 ± 48	73 ± 46***	57 ± 40	50 ± 37^†††^	89 ± 51
LVSV (mL)	90 ± 23	94 ± 23*	60 ± 15	58 ± 14^††^	101 ± 25
LVEF (%)	55 ± 11	59 ± 11***	55 ± 13	58 ± 13^†††^	55 ± 13
LVM (g)	135 ± 33	145 ± 38***	147 ± 38	151 ± 38^†††^	112 ± 33

Data are presented as mean ± SD. Significant differences are shown for MPS with the CZT gamma camera compared to the conventional gamma camera for Segment (*) and QGS/QPS (^†^) software, respectively, according to the convention *, ^†^*P* ≤ .05, ^††^*P* ≤ .01 and ***, ^†††^*P* ≤ .001

*LV*, left ventricular; *EDV*, end-diastolic volume; *ESV*, end-systolic volume; *SV*, stroke volume; *M*, mass; *MPS*, myocardial perfusion SPECT; *CZT*, cadmium-zinc-telluride; *CMR*, cardiac magnetic resonance

The MPS bias compared to CMR for all measures are shown in the “Appendix”.

## Discussion

This is the first study to explore the diagnostic performance of gated MPS with a CZT gamma camera compared to a conventional gamma camera for detection of MI and assessment of LV volumes and LVEF, using CMR as the reference method. Diagnostic accuracy is good for detection of MIs > 3% of the LV for MPS both with a CZT gamma camera and a conventional gamma camera. Furthermore, LV volumes are significantly underestimated with low precision by MPS compared to CMR but slightly less for the CZT compared to the conventional gamma camera, whereas the impact of MPS software on LV volume assessment is larger. The accuracy for assessment of LVEF is high.

The overall diagnostic accuracy for MPS with a CZT gamma camera for detection of MI was moderate. The overall sensitivity on a patient level was low/moderate but specificity was excellent. No difference was shown compared to MPS with a conventional gamma camera. The overall sensitivity was in line with two previous studies,^15,16^ although the specificity in the current study was higher. Compared to two other previous studies,^2,3^ the overall sensitivity in the current study was lower while specificity was equally high. However, compared to the study by Carlsson et al^3^ the MI size in the current study was smaller. For infarcts > 3% of the LV the sensitivity in the current study becomes higher. The main reason of why small infarcts is missed by MPS compared to CMR is the limited spatial resolution of MPS compared to CMR. The spatial resolution for the GE Discovery 530c camera was found to be approximately 7 mm and approximately 15 mm for a conventional gamma camera,^8^ whereas the spatial resolution for CMR is approximately 1.5 mm. Thus, a lower detection rate of small infarcts for MPS compared to CMR would be expected. Despite that the higher spatial resolution and count sensitivity of the CZT gamma camera would give a theoretical diagnostic advantage compared to a conventional gamma camera, no difference in diagnostic accuracy for MI detection between the CZT and conventional gamma camera was shown in the current study. As shown in Figure 2, there is an agreement of MI size assessed with CMR compared to MPS with the CZT as well as the conventional gamma camera which is in line with previous studies.^17,18^

It has been shown that patients with no clinical history of MI showing evidence of myocardial scar on CMR has an increased risk for future major adverse cardiac events (MACE).^19^ The patient group with smallest infarcts, mean infarct size of 1.4% of the left ventricle, had a > 7-fold increased risk for MACE. This highlights the importance of not only focusing on presence of stress-induced ischemia but also on MI when examining patients with MPS.

Recently, interesting results of myocardial fibrosis assessment have been published, using positron emission tomography (PET) and new radiotracers with ^68^Ga-labeled fibroblast activation protein inhibitor (FAPI). Post myocardial infarction imaging with ^68^Ga-FAPI-PET have shown fibroblast activation in the early phases of fibrosis evolvement, whereas no fibroblast activation was seen in mature myocardial scar.^20–22^ Further studies are warranted to elucidate the potential of using new radiotracers for myocardial fibrosis assessment in nuclear cardiology imaging.

LV volumes were significantly underestimated by MPS compared to CMR with both the CZT and the conventional gamma camera and both Segment and QGS/QPS MPS software, whereas LVM was significantly overestimated by MPS compared to CMR. Precision was low. Underestimation of LV volumes by MPS compared to CMR has also been shown in previous studies,^4–7,23^ and overestimation of LVM by MPS compared to CMR is in line with the results from a previous study.^7^ An important reason for this underestimation is the limited spatial resolution of MPS compared to CMR as discussed above. The thickness of a normal myocardial wall ranges approximately between 5 and 10 mm on CMR,^24^ which is in the same range as the limits for MPS spatial resolution. Delineation of myocardial wall contours is therefore challenging for MPS compared to CMR. Wall thickness on MPS is most often assessed larger than on CMR and the LV lumen therefore becomes smaller compared to CMR. Another reason why LV volumes are underestimated by MPS compared to CMR is differences in time resolution. In the current study, MPS was acquired with ECG-gating using 8 frames per cardiac cycle whereas the CMR images were acquired with 25 frames per cardiac cycle. It has been shown that assessment of LVEF by MPS using 8 frames per cardiac cycle gives an average reduction of LVEF by 3.7 percentage points compared to 16 frames per cardiac cycle.^25^

Assessment of LVEF by MPS showed good accuracy compared to CMR, with no bias for CZT MPS and a slight overestimation for conventional MPS compared to CMR. However, precision was low. A better accuracy for LVEF compared to LV volumes assessment by MPS has also been shown by previous studies.^4,5,7,23^ Since LVEF is calculated as a ratio including LVEDV and LVESV, an underestimation of LVEDV and LVESV of the same magnitude yields an accurate ratio, i.e., LVEF. In a previous study by Sharir et al,^26^ LVEF was found to be slightly higher for the CZT gamma camera compared to the conventional gamma camera. They examined patients post-stress with the conventional gamma camera first followed by CZT for all patients which may explain a systematic difference between the CZT and the conventional gamma camera due to post-ischemic stunning. Patients in the present study were examined at rest.

LV volumes by MPS differed significantly between the CZT and the conventional gamma camera but the absolute values of the differences were relatively small. Mean LVEDV and LVESV were significantly larger for the CZT gamma camera compared to the conventional gamma camera, thus closer to the reference values by CMR. This is probably due to the known higher spatial resolution and count sensitivity of the CZT gamma camera compared to the conventional gamma camera,^8^ which may facilitate a correct delineation of the myocardial walls, yielding a larger LV lumen.

Comparing MPS software, there were significant and relatively large differences between Segment and QGS/QPS. Bias for LVEDV and LVESV on MPS compared to absolute values by CMR were approximately 10%-15% for the Segment software and 40%-45% for the QGS/QPS software. However, LVEF by MPS showed no significant difference between the software, neither for the CZT nor for the conventional gamma camera. Compared to Hedeer et al^5^ and Soneson et al^7^ the bias for QGS/QPS software is in the same range, whereas the bias for Segment software was larger in the current study.

Hence, we recommend that absolute values of LV volumes and mass should be handled with care in a clinical report of an individual patient, and rather be interpreted compared to specific reference values for each setting, where type of gamma camera, image reconstruction parameters and MPS software have been taken into account. Since precision was low for all measures, caution should be taken so that methodological differences are not misinterpreted as biological differences and vice versa in a patient examined repeatedly.

## Limitations

The study population in the current study was relatively small. Thus, a small difference in diagnostic performance between the gamma cameras for detection of MI might be missed. However, given that there were no differences between the gamma cameras in overall sensitivity, specificity, PPV and NPV for detection of MI, the likelihood for type 2 errors is considered to be small. The diagnostic accuracy for MPS found in the current study is partly dependent on the size of the infarcts in the study population. In the current study a significant number of patients had small sized infarcts. The number of patients with MI in the current study population was high, and probably higher than the prevalence to be expected in patient populations of many MPS diagnostic centers. Of note, this does not affect the sensitivity or specificity. On the other hand, the use and availability of MPS are probably highly variable for different diagnostic centers worldwide, with a large variation in pre-test probability in the patients referred for MPS. Vessel territories were divided into two territories, LAD territory and LCx/RCA territory. The reason why LCx and RCA territories were analyzed as one territory is the difficulties to correctly determine the LCx and RCA territories.^27^ In delineation of the left ventricle in the CMR images, trabecular and papillary muscles not contiguous with the myocardial wall were excluded, thus included in the LV cavity volume. This may lead to an over estimation of the LV cavity blood volumes by CMR by approximately 10%, thus partly explaining the differences in assessed LV volumes between MPS and CMR.^28^ The MPS images were interpreted by visual analysis only and not automatically quantified by the MPS software.

## Conclusion

Differences in diagnostic accuracy for detection of MI and assessment of LV volumes and LVEF between MPS with a CZT gamma camera compared to a conventional gamma camera, are small and do not appear to be clinically significant.

## New Knowledge Gained

The diagnostic performance of gated MPS with a CZT gamma camera compared to a conventional gamma camera for detection of MI and assessment of LV volumes and LVEF, using CMR as the reference method, shows good diagnostic accuracy for detection of MIs > 3% of the LV with both gamma cameras. However, LV volumes are significantly underestimated by MPS compared to CMR but slightly less for the CZT compared to the conventional gamma camera, whereas the impact of MPS software is larger.

### Supplementary Information

Below is the link to the electronic supplementary material.

Supplementary file1 (PPTX 376 kb)

## Figures and Tables

**Figure 4:**
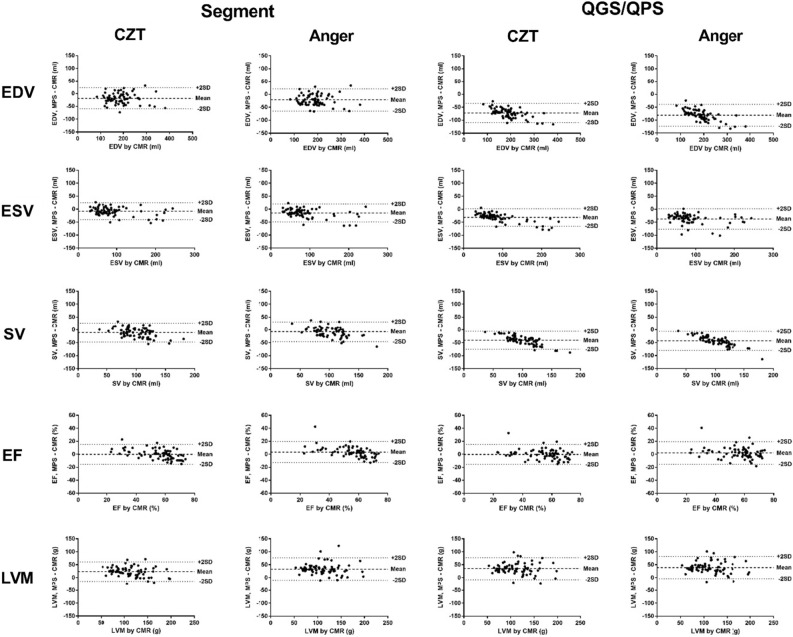
The absolute difference between MPS and CMR for EDV, ESV, SV, EF and LVM compared to CMR. Separate plots are shown for MPS with a traditional and a CZT gamma camera and with the MPS software QGS/QPS and Segment

## Appendix

See Figure 4.

## Abbreviations

MPS: Myocardial perfusion single-photon emission computed tomography
CZT: Cadmium-zinc-telluride
CMR: Cardiac magnetic resonance
LGE: Late gadolinium enhancement
LV: Left ventricle
EF: Ejection fraction
MI: Myocardial infarct
