# Supplementary material for: Diagnostic accuracy for CZT gamma camera compared to conventional gamma camera technique with myocardial perfusion single-photon emission computed tomography: Assessment of myocardial infarction and function
Source: J Nucl Cardiol. 2023 Mar 13;30(5):1935–46. doi: 10.1007/s12350-022-03185-0 (PMC10558368; doi:10.1007/s12350-022-03185-0)
Supplement: Supplementary file 1 — Supplementary file1 (PPTX 376 kb) [file 12350_2022_3185_MOESM1_ESM.pptx]

## Slide 1
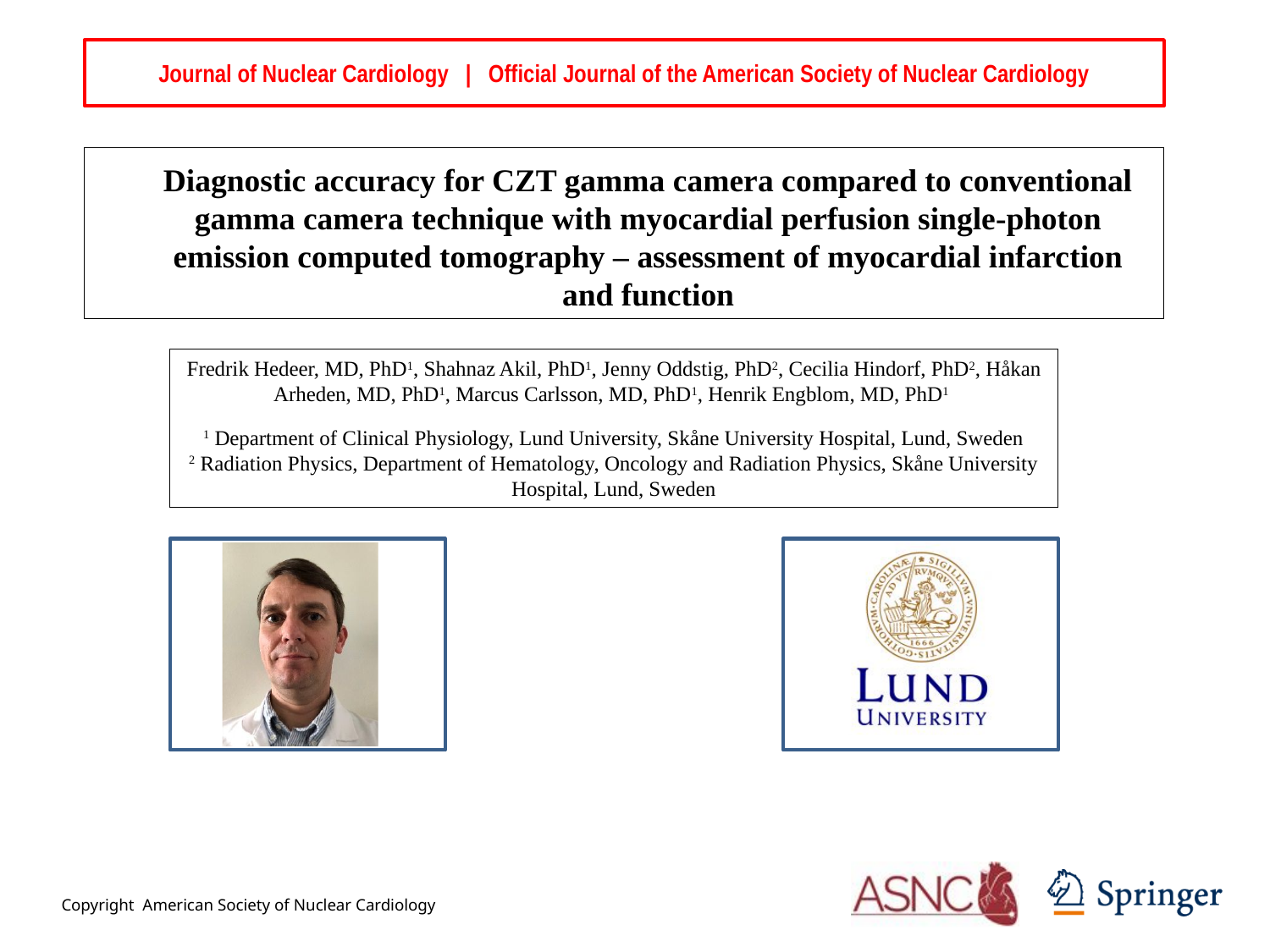

Journal of Nuclear Cardiology | Official Journal of the American Society of Nuclear Cardiology
# Diagnostic accuracy for CZT gamma camera compared to conventional gamma camera technique with myocardial perfusion single-photon emission computed tomography – assessment of myocardial infarction and function
Fredrik Hedeer, MD, PhD1, Shahnaz Akil, PhD1, Jenny Oddstig, PhD2, Cecilia Hindorf, PhD2, Håkan Arheden, MD, PhD1, Marcus Carlsson, MD, PhD1, Henrik Engblom, MD, PhD1
1 Department of Clinical Physiology, Lund University, Skåne University Hospital, Lund, Sweden
2 Radiation Physics, Department of Hematology, Oncology and Radiation Physics, Skåne University Hospital, Lund, Sweden
Copyright American Society of Nuclear Cardiology

## Slide 2
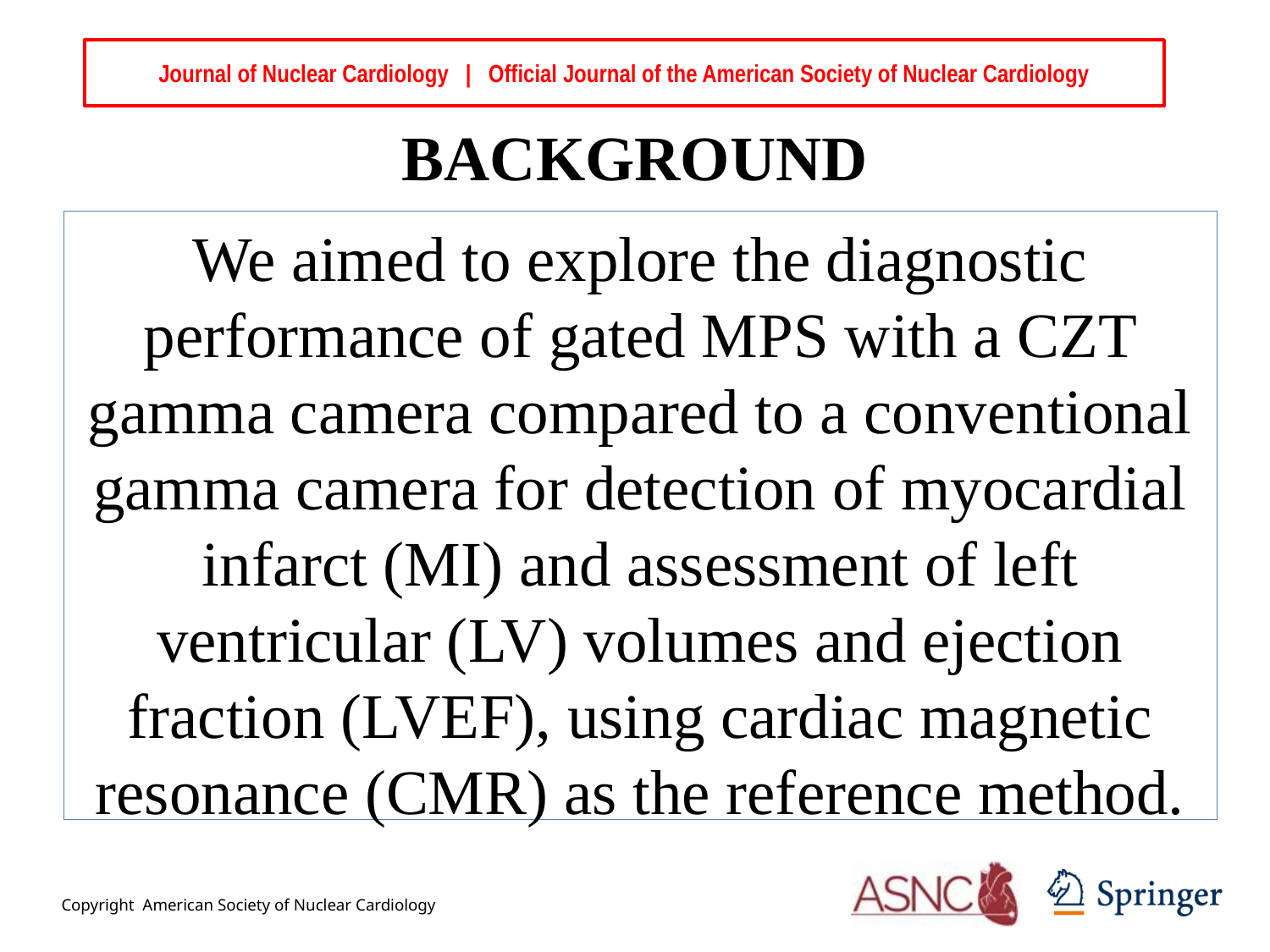

Journal of Nuclear Cardiology | Official Journal of the American Society of Nuclear Cardiology
# BACKGROUND
We aimed to explore the diagnostic performance of gated MPS with a CZT gamma camera compared to a conventional gamma camera for detection of myocardial infarct (MI) and assessment of left ventricular (LV) volumes and ejection fraction (LVEF), using cardiac magnetic resonance (CMR) as the reference method.
Copyright American Society of Nuclear Cardiology

## Slide 3
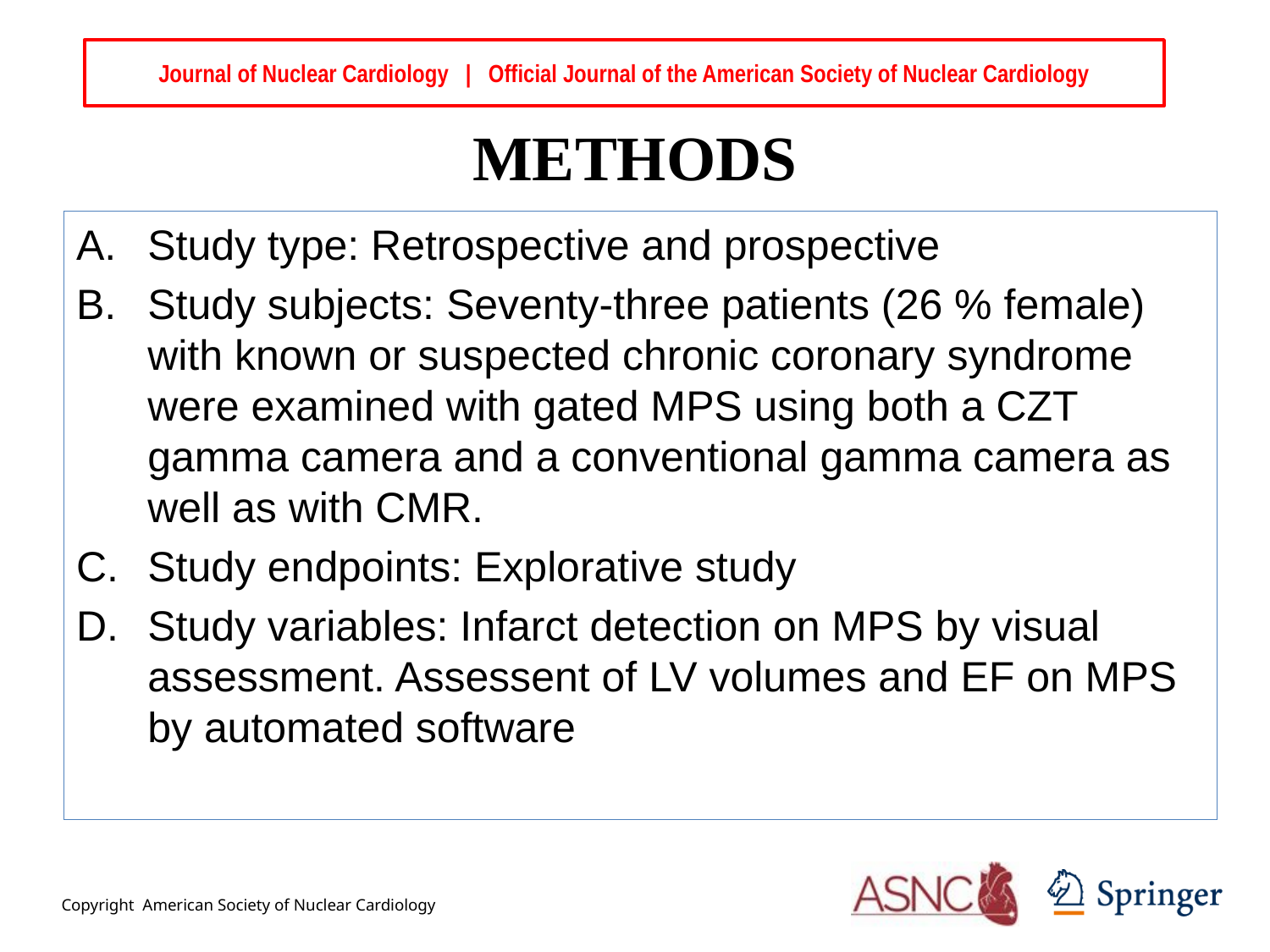

Journal of Nuclear Cardiology | Official Journal of the American Society of Nuclear Cardiology
# METHODS
Study type: Retrospective and prospective
Study subjects: Seventy-three patients (26 % female) with known or suspected chronic coronary syndrome were examined with gated MPS using both a CZT gamma camera and a conventional gamma camera as well as with CMR.
Study endpoints: Explorative study
Study variables: Infarct detection on MPS by visual assessment. Assessent of LV volumes and EF on MPS by automated software
Copyright American Society of Nuclear Cardiology

## Slide 4
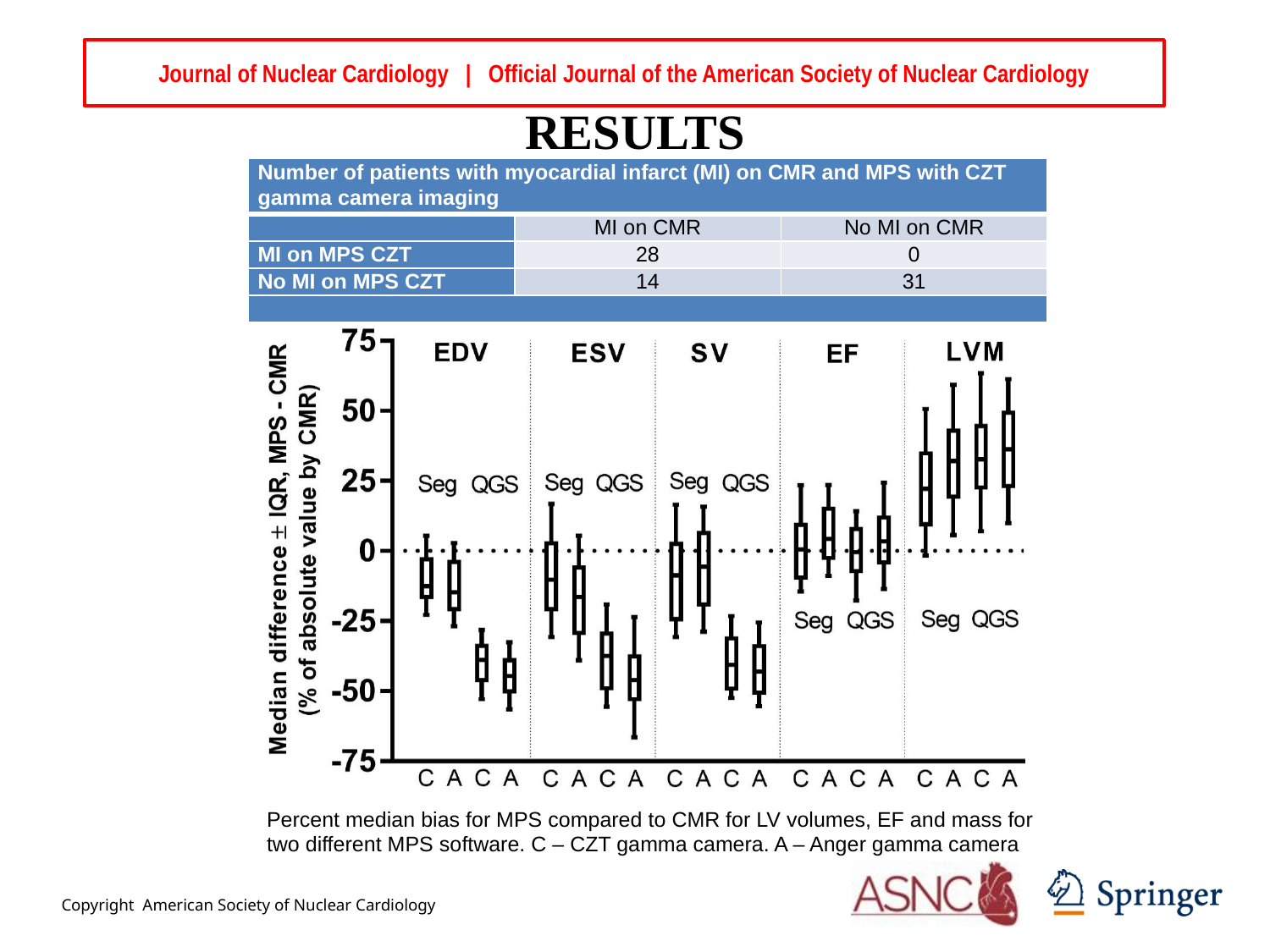

Journal of Nuclear Cardiology | Official Journal of the American Society of Nuclear Cardiology
# RESULTS
| Number of patients with myocardial infarct (MI) on CMR and MPS with CZT gamma camera imaging | | |
| --- | --- | --- |
| | MI on CMR | No MI on CMR |
| MI on MPS CZT | 28 | 0 |
| No MI on MPS CZT | 14 | 31 |
| | | |
Percent median bias for MPS compared to CMR for LV volumes, EF and mass for two different MPS software. C – CZT gamma camera. A – Anger gamma camera
Copyright American Society of Nuclear Cardiology

## Slide 5
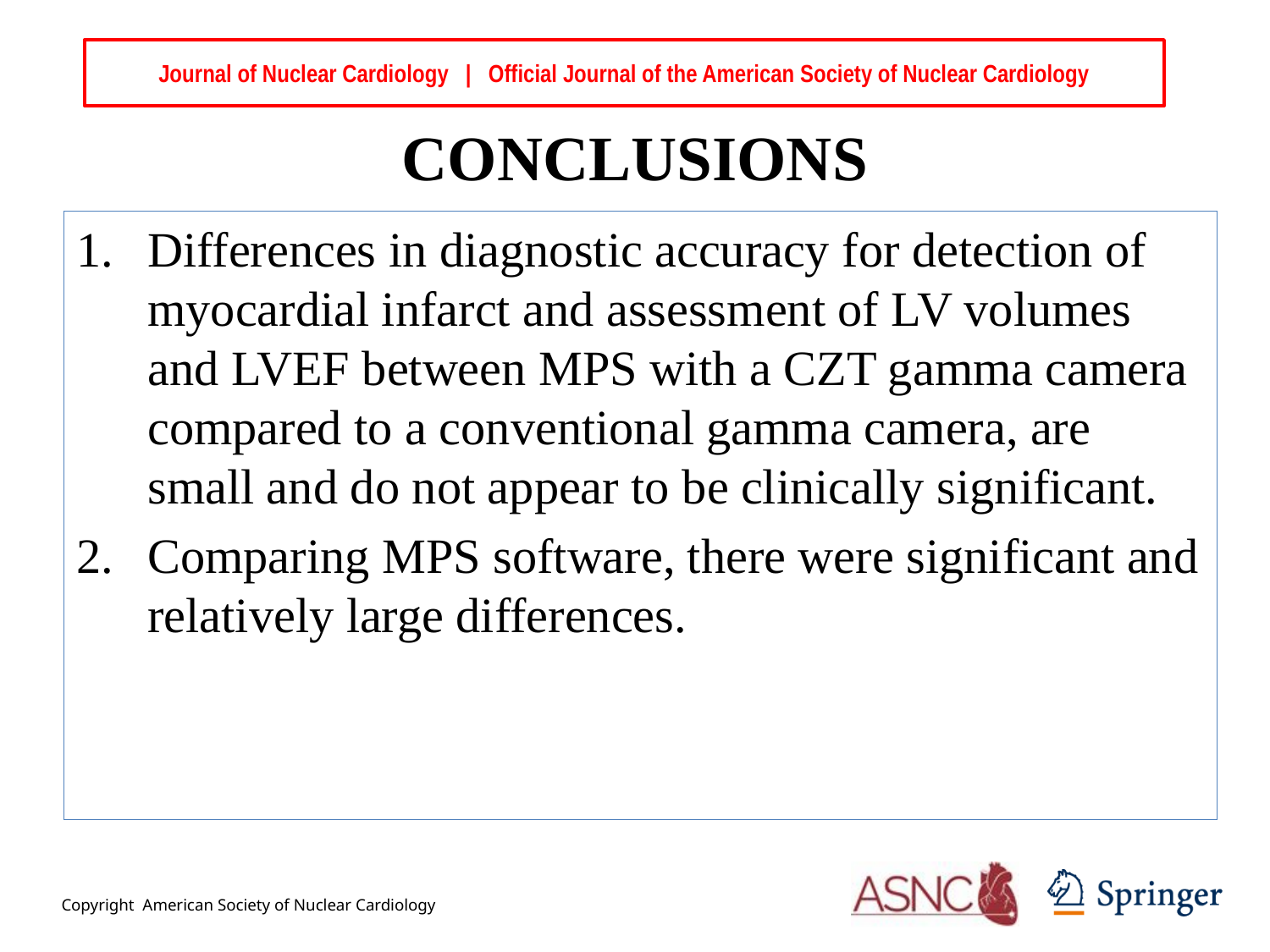

Journal of Nuclear Cardiology | Official Journal of the American Society of Nuclear Cardiology
# CONCLUSIONS
Differences in diagnostic accuracy for detection of myocardial infarct and assessment of LV volumes and LVEF between MPS with a CZT gamma camera compared to a conventional gamma camera, are small and do not appear to be clinically significant.
Comparing MPS software, there were significant and relatively large differences.
Copyright American Society of Nuclear Cardiology
